# Cholera amidst COVID-19 pandemic: African healthcare system in jeopardy

**DOI:** 10.31744/einstein_journal/2022CE6938

**Published:** 2022-04-25

**Authors:** Sude Çavdaroğlu, Irem Aktar, Mohammad Mehedi Hasan, Ana Carla dos Santos Costa, Abdullahi Tunde Aborode, Shoaib Ahmad, Mohammad Yasir Essar

**Affiliations:** 1 Maltepe University Istanbul Turkey Maltepe University, Istanbul, Turkey.; 2 Istanbul University Istanbul Turkey Istanbul University, Istanbul, Turkey.; 3 Department of Biochemistry and Molecular Biology Faculty of Life Science Mawlana Bhashani Science and Technology University Tangail Bangladesh Department of Biochemistry and Molecular Biology, Faculty of Life Science, Mawlana Bhashani Science and Technology University, Tangail, Bangladesh.; 4 Faculdade de Medicina Universidade Federal da Bahia Salvador BA Brazil Faculdade de Medicina, Universidade Federal da Bahia, Salvador, BA, Brazil.; 5 Healthy Africans Platform, Research and Development Ibadan NG Nigeria Healthy Africans Platform, Research and Development, Ibadan, NG, Nigeria.; 6 Punjab Medical College Faisalabad Pakistan Punjab Medical College, Faisalabad, Pakistan.; 7 Medical Research Center Kateb University Kabul Afghanistan Medical Research Center, Kateb University, Kabul, Afghanistan.

Dear Editor,

Cholera is a well-known disease, caused by the toxin of the bacterium *Vibrio cholerae,* and has resulted in severe problems to the health systems of African countries, over the years, even after more than 50 years after its resurgence, in 1970. Among the nearly 1.4 to 4.0 million cases, and 21,000 to 143,000 deaths per year worldwide, most of the burden occurs in African countries, especially in Sub-Saharan Africa.^(1)^

The cholera in Africa occurs in two settings, as an endemic situation that happens every 2 to 3 years, in certain seasons, and as an epidemic situation that starts acutely and threatens thousands of lives, while burdening the healthcare systems.^(2)^Between 2015 and 2019, ten cholera outbreaks affected Sub-Saharan African countries: Democratic Republic of Congo (DRC) in 2019; Ethiopia in 2019 and 2017; Kenya from April to June 2015; Mozambique in April 2019; South Sudan in 2017; Tanzania in August 2015; Zambia in October 2017; Zanzibar in 2016; and Zimbabwe in 2018. Some of these outbreaks reached between 4,000 and 7,000 cases of cholera.^(3)^

In Africa, the recurrent and continuous cholera outbreaks have been associated mainly with overcrowding, water supply and sanitation problems, and poverty.^(4)^ Climate change and environmental reservoirs also take a toll on the cholera and ease the transmission of disease. Transmission also becomes easier especially in crowded life settings, such as during wars, in prison and refugees camps.^(5)^ Therefore, some African issues, such as cross-border migration, socioeconomic factors, and political instability, aggravate the situation.^(6)^

The preventive actions against cholera are based on improving sanitary conditions and hygiene. The World Health Organization (WHO) further advocates for the use of oral cholera vaccines (OCV) as a transient protection, since vaccines have been proven to be effective to combat cholera especially during an epidemic situation.^(7)^ Despite the efforts put into by the local governments, and the WHO initiatives to eliminate the disease, by launching the Global Task Force on Cholera Control, there are many obstacles hindering massive and proactive OCV use in Africa, and vaccination is still not at the expected levels. The success of the OCV program depends on huge financial investment, which is essential to build an immunization infrastructure, including vaccine logistics and transportation. Modeling data were also not able to bring a concrete result for the beneficiary of vaccine campaigns.^(8)^

As expected, the coronavirus disease 2019 (COVID-19) pandemic had a negative impact on the implementation of preventive measures, and the epidemiological control of numerous infectious diseases around the world.^(9,10)^Among African countries, the situation was no different. Poor surveillance systems got worse during the pandemic, which affected viral diseases, such as measles and yellow fever.^(11,12)^Based on empirical evidence, COVID-19 has severely impacted the preventive and control measures of cholera. However, the actual extent of the impact is not yet fully known, given lack of an efficient surveillance system.^(4)^ Data available from the first weeks of 2021 showed that West and Central Africa have recorded fewer cases of cholera. However, the case fatality rate at week 5, in 2021, is the highest in the last 3 years, and can be further explained by the underestimated impacts of the COVID-19 pandemic due to saturation of health facilities, reluctance to go to health facilities when presenting the first symptoms, and overload of surveillance services.^(13)^

It is undeniable that the pandemic has created a fertile environment for future outbreaks. Both the healthcare settings and healthcare professionals are overburdened with the pandemic. This overload has significantly reduced the time devoted to non-COVID-19 patients. Shortly after the first cases were seen, over 6 million people were under the threat of losing sanitation, which is a major concern for a possible cholera outbreak.^(14)^

Coronavirus disease 2019 has also reduced health-seeking behavior, because of the increasing fear of being infected by the virus. This fear is attributed to lack of adequate resources, such as personal protective equipment, medicines, and proper sanitary conditions, as well as decreased immunization against cholera, and the major focus being the pandemic at hand.^(14)^Suspension of the fight against cholera, together with shortages in food and water, and displacements across the country have already caused an increase in cases in Ethiopia.^(15)^

Before the COVID-19 pandemic, 47 African countries adopted the Regional Framework for the implementation of the global strategy of cholera prevention and control. The countries in line with the framework have committed themselves to limit the spread of cholera by taking evidence-based actions, improving treatment strategies, tightening border surveillance, as well as promoting use of OCV with increasing investments in water supplies.^(16)^ However, the pandemic had a negative impact on several strategies that would be adopted, such as decline in the provision of water, sanitation and hygiene (WASH) services, vaccine supplies, vaccine campaigns, and humanitarian aid, which were harmed due to measures of isolation and social distance, to prevent contagion against the coronavirus.^(17)^

To mitigate the situation and avoid future outbreaks, a scientific approach and surveillance programs for cholera, to understand the epidemiology and detect risky areas, are essential to manage prevention and spread of the diseases. The Centers for Disease Control and Prevention (CDC), have a public database of cholera cases. However, the true number of cases is underestimated, which makes it difficult to take measures appropriate to the actual epidemiological situation.^(3)^Therefore, it is necessary to improve laboratory and epidemiological surveillance systems. Moreover, the provision of WASH services and commodities is essential to prevent infection during the outbreak of infectious diseases, including cholera. In addition, to improve cholera control, it is necessary to improve health systems capacity to diagnose the disease, since inconsistencies in case definitions and lack of laboratory diagnostic also contribute to underreporting.

Even during the COVID-19 pandemic, it is necessary to reinforce the need to improve the multifactorial approach, as a key to prevent and control cholera outbreaks in Africa. It is undeniable that only with effective and continuous surveillance, vaccination, water, sanitation, and hygiene programs, associated with social mobilization and treatment, will African countries be able to provide a rapid and effective response to cholera outbreaks.

In conclusion, cholera is responsible for major outbreaks in Africa. Hygiene is crucial for both cholera and coronavirus transmission. Adhering to cholera prevention commitments alongside an efficient surveillance system, to track cholera outbreaks and to detect public health needs towards combating cholera in Africa, is the essential need in the current pandemic.
